# School Based Tooth Brushing and Annual Silver Diammine Fluoride Application as a Highest Priority Package for Achieving Universal Oral Health Care for Cambodian Children

**DOI:** 10.3389/froh.2021.667867

**Published:** 2021-05-07

**Authors:** Bathsheba Turton, Jilen Patel, Chanthyda Sieng, Ranuch Tak, Callum Durward

**Affiliations:** ^1^Faculty of Dentistry, University of Puthisastra, Phnom Penh, Cambodia; ^2^Department of Pediatric Dentistry, University of Western Australia, Perth, WA, Australia; ^3^Healthy Kids Cambodia Project, One-2-One Charitable Trust, Phnom Penh, Cambodia

**Keywords:** silver diammine fluoride, universal health coverage, highest priority package, daily toothbrushing, school health

## Abstract

**Background:** Achieving Universal Oral Health Care among Low-to Middle-Income settings is challenging and little literature exists around exploring what a “Highest Priority Package” of care might look like in the context of oral health. The Healthy Kids Cambodia (HKC) program differs from most conventional school dental services in that the initial package of care that is offered is daily toothbrushing with 1,500 ppm fluoride toothpaste (DTB) together with the topical application of Silver Diamine fluoride (SDF) for management of lesions in primary teeth.

**Aim:** To examine tooth level outcomes for 8- to 10-year old children from two schools that performed DTB with application of SDF at differing time-points.

**Design:** This was an observational cohort study that examined lesion progression among children in late mixed dentition at two schools. Data were collected using the dmft and pufa indices. Both schools received materials and training for DTB at baseline. School One received SDF at baseline while School Two received SDF after 9-months. Intraoral examinations were performed and the presentation of primary teeth with cavitated carious lesions were compared at baseline and 12 m. If a tooth was still caries-active or had become pulpally involved, this was considered to be an unacceptable outcome. Descriptive analysis was performed the chi-squared test was used to examine differences in the proportion of teeth with unacceptable outcomes by school membership.

**Results:** Of the 521 children recruited, 470 (90.2%) were followed. Where there was a delay in SDF application (School 2) there was a three times greater chance of an unacceptable outcome. Ten percentage of primary teeth in School One and 33% of primary teeth in the School Two had unacceptable outcomes.

**Conclusion:** The present study offers data on expected effect sizes that might inform future step-wedged clinical trials to validate an oral health Highest Priority Package of care for Cambodian children. The delivery of a package of care that includes both DTB and SDF can prevent adverse outcomes, such as dental infections, in primary teeth with carious lesions.

## Introduction

Dental caries is a disease which is resource-intensive to treat. In addition, conventional surgical management of the disease with injections and drills can be challenging for children to accept [1]. In 2002 the WHO described the Basic Package of Oral Care (BPOC) as a way of achieving universal oral health care [2]. This BPOC was a first step in describing a package of care that might be an appropriate vehicle for achieving Universal Access to Oral Health care in low resource settings. The WHO defines Universal Health Coverage (UHC) as a situation in which “all people have access to the health services they need, when and where they need them, without financial hardship” [3]. One of the challenges in the delivery of UHC is to set priorities, to define who gets what and under which circumstances. By doing this, interventions can tested to explore value for money, their ability to address or reduce a significant disease burden and their feasibility in Low and Middle Income Country (LMIC) settings. Those principals are used to define what might be included in an Essential Universal Health Coverage (EUHC) or a Highest Priority Package (HPP).

The BPOC was proposed as an EUHC and comprises: OUT (Oral Urgent Treatment), ART (Atraumatic Restorative Treatment), and AFT (Affordable Fluoride Toothpaste). A number of countries have made the BPOC part of their policies, and although those interventions have been shown to be clinically effective as individual therapies, there is not a lot of evidence around the cost of delivery or the effectiveness of the package, delivered at population level. Cambodia was one of those countries where the BPOC has been adopted, at least in part, for the delivery of limited oral health services by dental nurses in community health centers. The study by Chher et al. cfound significant challenges around the ongoing supply of instruments and materials [4].

It is well-documented that Cambodian school children have a severe burden of dental caries; the mean dmft for 6-year-old children is 9-teeth, and yet <5% of children have received any restorative care [5]. In 2016 a program called Healthy Kids Cambodia (HKC) was implemented in several schools. It includes several components of the BPOC and it also adds the use of Silver Diammine Fluoride (SDF) as an important therapeutic intervention. This contrasts with the regional (Fit for Schools) FFS program which promotes daily toothbrushing with 1,500 ppm fluoride toothpaste and daily handwashing in primary schools. The FFS program demonstrated a 17% reduction in new carious lesions among pilot schools. However, this approach did not significantly reduce the incidence of pulpally-involved teeth and did not address the high prevalence and severity of existing disease [6].

As part of establishing an integrated approach to disease management, the HKC program built on what had been learned in the FFS program. It added a novel triage system (Table 1) that helps to distribute resources, for preventive care as well as restorative and non-restorative management of carious lesions [7]. There are two key issues with upscaling the HKC three levels of care at scale; the first is that it may be unaffordable to deliver the full program, and the second is that there may be insufficient workforce to perform all of the interventions. The first level of care costs $3 per child to deliver and this paper aims to evaluate the clinical outcomes of carious lesions in the primary dentition of children in the late mixed dentition from two schools that started the HKC project at different time points of the 2017–2018 academic year.

**Table 1:** The three-tiered system used to triage children based on clinical presentation and treatment needs.

**Referral**	**Triage criteria**	**Treatments**
Level 1	All participants	General health screening where possible.
		Daily tooth brushing with fluoridated toothpaste and hand washing.
		Biannual basic health screening, de-worming, and Vitamin A supplementation. Silver diammine fluoride application to arrest caries in primary teeth.
		Oral health education.
Level 2^a^	All children 6- to 8-years of age AND older children who have open cavitations on permanent posterior teeth	Placement of restorations using the atraumatic restorative technique (ART) and pit and fissure sealants in permanent teeth using glass ionomer cement.
Level 3^a^	Children with active (acute) infection OR those who have open cavitation on permanent anterior teeth OR those who have cavitations on permanent posterior teeth that are not restorable by ART	Comprehensive oral rehabilitation in a fixed or mobile dental clinic, including extractions and conventional restorations.

a *Children may qualify both level 2 and level 3 depending on their clinical presentation. These categories are not mutually exclusive*.

## Methods

This study examined caries lesion progression in two different schools in the HKC project, both schools were in Phnom Penh city and will be referred to as “School One” and “School Two.” Care was delivered to those schools through a collaboration between the Faculty of Dentistry at the University of Puthisastra, the New York University College of Dentistry Global Student Outreach Program (NYU Dentistry), and the Non-Governmental Organization (NGO) One-2-One Cambodia. All children receiving care went through routine consent processes that involved delivery of information to parents and teachers, and an opt-out process. Children and parents were free to refuse participation at any point during the program without consequence. Ethical approval for this study was received from the National Ethics Committee for Health Research, Ministry of Health, Cambodia.

### Clinical Interventions

**Figure 1:**
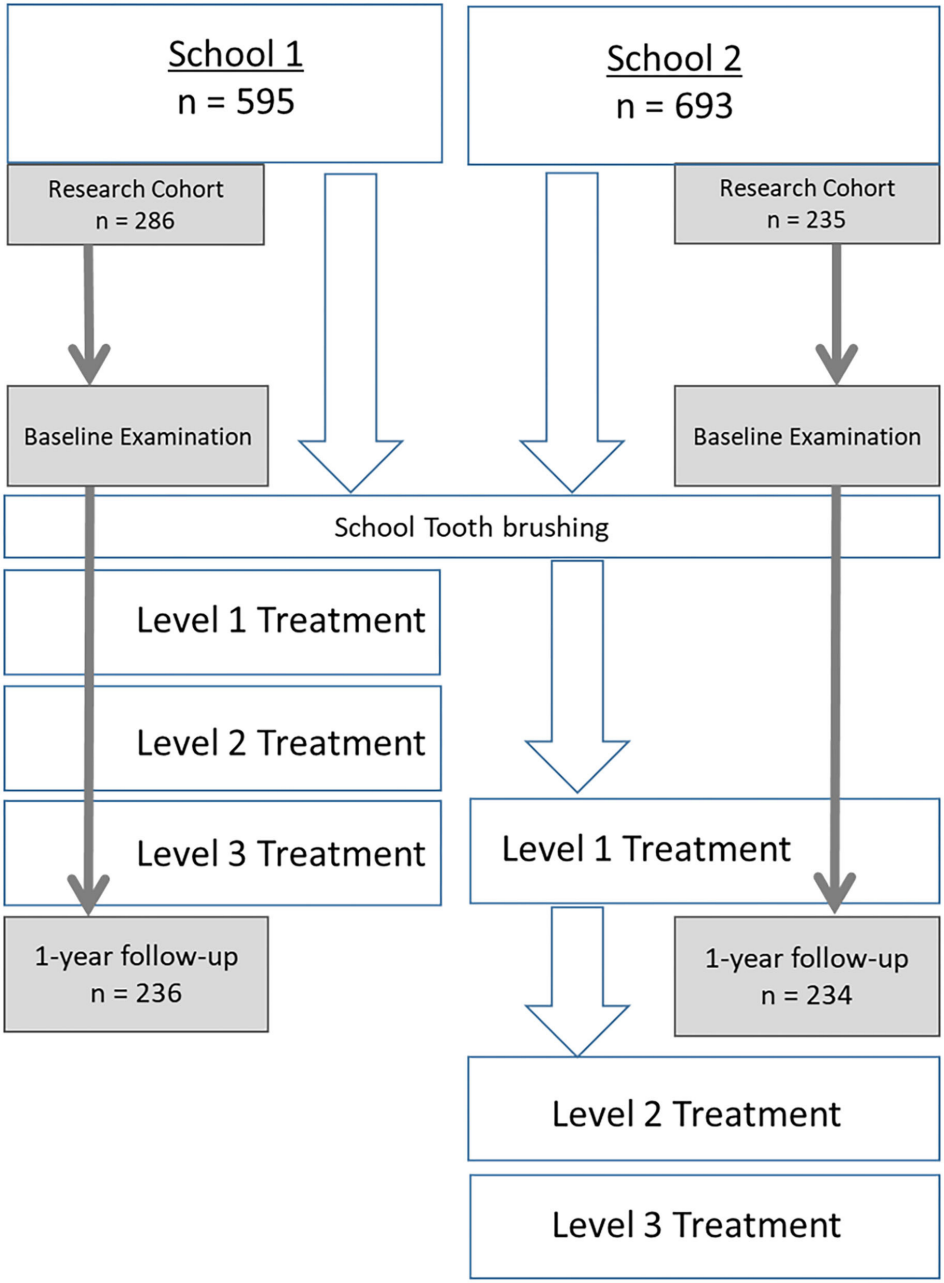
Project flow of events.

Both schools were scheduled to start the HKC program at the beginning of the 2017–2018 academic year and so both schools were given training and resources to facilitate daily tooth brushing with fluoride toothpaste (1,500 ppm) at the beginning of the study. At baseline, both schools also received training and materials for DTB and consenting children in Grade 3 and Grade 4 at both participated in detailed clinical examinations at baseline. School 1 also received Level 1 care. School received level 1 care after 9 months. Figure 1 describes the progression of activities in each school across the time-line. SDF was applied to eligible carious primary teeth by trained dental students under the supervision of the UP community outreach clinical supervisor (an experienced dentist).

### Clinical Examination

Children were examined at school in a supine position using handheld torches for illumination at both baseline in June 2017 and at follow-up in July 2018. The WHO Basic Methods for Dental Surveys was used as the guideline for recording dmft index data [8] and the pufa index [9]. One of three examiners examined the children at the two time points. Examiners underwent 4 h of training prior to each data collection phase and were calibrated (Interclass Correlation Coefficient >0.85 for inter-examiner reliability) against the principal investigator (BT). For primary teeth with cavitated carious lesions not involving the pulp additional data were recorded on the color and hardness of the lesions at follow-up. Hardness was assessed using a 3-point scale based on the texture (soft, leathery, or hard) of the lesion when probed using a ball-ended probe. The color of the lesion was defined across a 4-point scale including yellow, brown, mixed or black.

### Data Management and Analysis

Data were entered into an Excel spreadsheet and then transferred into SPSS Version 23 (IBM SPSS Inc., Chicago, IL, USA) for analysis. Data were analyzed descriptively to examine the sociodemographic and clinical characteristics of participants as well as clinical characteristics of teeth. Data were re-categorized to create the key outcomes of interest. Arrest was defined as those lesions which were coded as both “hard” in texture or “black” in color. A lesion was considered to be “caries active” if it had a texture that was soft or leathery and a color that was not black. A “pulpally involved” tooth was defined as any tooth which met any of the four PUFA criteria. The category of “active infection” was based on a tooth meeting the criteria of “fistula” or “abscess” of the PUFA index. The category of a “new pulpally involved tooth” was defined as any tooth that progressed from initially having a carious lesion (as described above) but no PUFA score, to a tooth manifesting any of the four PUFA categories.

The tooth was considered to have an “acceptable outcome” if: (a) the caries was “arrested” and there was no evidence of pulpal involvement, or (b) if it had exfoliated during the past 12 months. An unacceptable outcome was defined as a tooth where the caries was still active or where the lesion had advanced to one of the PUFA categories. The Chi-squared-test was used to examine differences in outcome by school membership.

## Results

Of the 521 participants, at baseline most (319; 61.2%) children had one or more pulpally involved teeth present. Children in this study were in the late mixed dentition, and the mean dmft was 3.0 (SD 2.9). There was a statistically significant (*P*-value = 0.001) difference in age whereby the children at School 2 (8.8; SD 0.9) were younger than those at School 1(9.0; SD;1.1); the mean age as 9.0 (SD 1.0). There was a significant difference in the proportion of children with one or more teeth which were pulpally-involved at baseline between School 1 (54.2% of teeth) and School 2 (69.8% of teeth) (Table 2).

**Table 2:** Sociodemographic characteristics and referral qualification of participants by clinical characteristics at baseline.

	***n* (%)^a^**	**Any fistula/abscess** ***n* (%)**	**Presence of one or more pulpally involved teeth *n* (%)**	**dmft Mean (SD)**
School 1	286 (54.9)	4 (1.4)	155 (54.2)^b^	2.6 (2.6)^c^
School 2	235 (45.1)	3 (1.3)	164 (69.8)^b^	3.7 (3.1)^c^
**Sex**				
Male	264 (50.7)	4 (1.5)	161 (61.0)	3.2 (3.0)
Female	257 (49.3)	3 (1.2)	158 (61.5)	2.9 (2.8)
Overall	521 (100.0)	7 (1.3)	319 (61.2)	3.0 (2.9)

a *Brackets contain column percent*.

b *Difference between school is statistically significant (P-value < 0.05; chi-square test)*.

c *Difference between school is statistically significant (P-value < 0.05; Kruskal-Wallis test)*.

A total of 470 children were followed through the study (90.2% follow-up). Those children belonging to School Two had a statistically significantly higher rate of follow-up (99.6% for School 2 vs. 82.5% for School 1) (Table 3).

**Table 3:** Attrition of participants between baseline and 1-year follow-up.

	**Followed**	**Lost**	**Total**
	***n* (%)**	***n* (%)**	***n* (%)^a^**
**Age-group**			
7-years	3 (100.0)	0 (0.0)	3 (0.6)
8-years	110 (89.4)	13 (10.6)	123 (23.6)
9-years	159 (33.8)	12 (23.5)	171 (32.8)
10-years	129 (86.0)	21 (14.0)	150 (28.8)
11-years	50 (94.3)	3 (5.7)	53 (10.2)
12-years	15 (93.8)	1 (6.2)	16 (3.1)
13-years	2 (66.7)	1 (33.3)	3 (0.6)
14-years	2 (100.0)	0 (0.0)	2 (0.4)
**Sex**			
Male	236 (89.4)	28 (10.6)	264 (50.7)
Female	234 (91.1)	23 (8.9)	257 (49.3)
**School membership** ^b^			
School 1	236 (82.5)	50 (17.5)	286 (54.9)
School 2	234 (99.6)	1 (0.4)	235 (45.1)
Total	470 (90.2)	51 (9.8)	521 (100.0)

a *Brackets contain column percentage*.

b *P-value ≤ 0.05; chi squared test for differences in proportions among groups within the same column*.

A total of 1,391 teeth with caries lesions were observed at baseline and 1,233 (88.6%) were followed (Table 4). Most of the teeth (76.6%) were molars and around half (46.7) were pulpally involved. There was a significant (*P* ≤ 0.001; chi squared-test) difference in loss-to follow-up by school where by teeth belonging to children from school 1 were more likely to be lost to follow-up.

**Table 4:** Attrition of teeth between baseline and 1-year follow-up by clinical characteristics.

	**Followed**	**Lost**	**Total**
	***n* (%)**	***n* (%)**	***n* (%)^a^**
**Pulpal involvement**			
Pulpally involved	574 (88.4)	75 (11.6)	649 (46.7)
No pulpal involvement	659 (88.8)	83 (11.2)	742 (53.3)
**Tooth type**			
Molar	942 (88.5)	123 (11.5)	1,065 (76.6)
Incisor	291 (89.3)	35 (10.7)	326 (23.4)
**School membership** ^b^			
School 1	497 (76.0)	157 (24.0)	654 (47.0)
School 2	736 (99.9)	1 (0.01)	737 (53.0)
Total	1,233 (88.6)	158 (11.4)	1,391 (100.0)

a *Brackets contain column percentage*.

b *P-value ≤ 0.05; chi squared test for differences in proportions among groups within the same column*.

**Figure 2:**
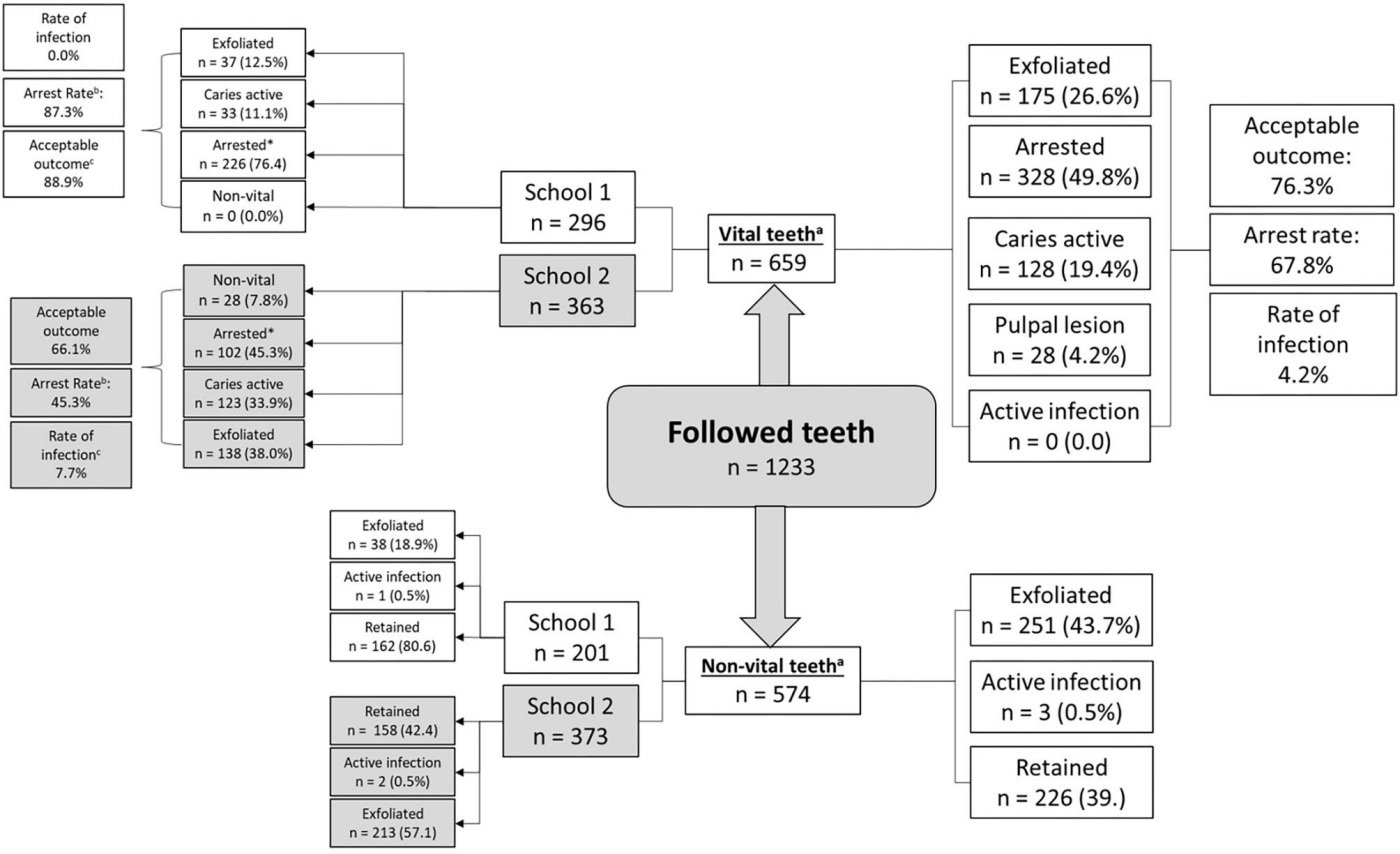
Tooth-level outcomes by baseline lesion description and school membership. ^a^*P*-value for difference in the proportion of cavitated and pulpally involved lesions ≤ 0.001; chi squared test. ^b^*P*-value for difference in arrest rate by school ≤ 0.001; chi squared test. ^c^*P*-value for difference in rate of infection by school membership ≤ 0.001; chi squared test.

Figure 2 presents data on tooth-level outcomes after 1 year. Of those caries lesions that were not pulpally involved at baseline, 76.3% had an acceptable outcome irrespective of school membership. Children in School 1 had a significantly higher rate of arrest and a higher chance of an “acceptable outcome” after taking into account metrics for both caries arrest and exfoliation; one in three teeth with carious lesions at baseline had an unacceptable outcome at follow up at School 2 vs. only 1 in 10 teeth at School 1. None of the cavitated teeth treated with SDF at baseline (School 1) became pulpally involved, whereas 7.7% of the teeth where treatment had been delayed by 9 months became pulpally involved (School 2).

The 28 new pulpally involved teeth represent 20 children (8.5% of those in school 2) who experienced the 9-month delay in receiving the SDF treatment. Notably, none of the teeth which received SDF at baseline (School One) had dental caries which progressed to involve the pulp after 12 months.

## Discussion

The present study observed tooth level outcomes among children in two Cambodian primary schools who had received DTB and SDF. While the evidence for DTB as part of an Essential Health Package (such as FFS) for primary school children is well-established in the global literature, this study starts to build evidence around the benefits of the timely application of SDF to avoid unnecessary suffering as the result of caries involving the dental pulp. The study found that where SDF was applied at baseline, after 12 months there were no new primary teeth with pulpal involvement and that 9 out of 10 teeth had an acceptable outcome. Where SDF treatment was delayed, 1 in 10 children exhibited pulpal complications in one or more primary teeth and only 7 in 10 teeth had acceptable outcomes. This demonstrates the benefit of SDF as an early intervention therapy for managing carious lesions among a high caries population. This study is unique in that it examines the combination of DTB and SDF together as an appropriate and effective package to reduce the incidence of pulpally involved lesions in primary teeth among children in the late-mixed dentition.

Before further discussion it is appropriate to point out the limitations of the study, most of which originate from the sample selection. The participants in this study were selected based on convenience sampling and there were differences in the proportions lost to follow-up and demographics of the schools; the present study was not able to account for these differences. That means that while the study was able to examine clinical experiences among a particular age-cohort, it comes with a high chance of bias and that limits the generalizability of findings. It is also important to point out that while the proportion of active infections (defined as teeth with a fistula or abscess using the PUFA index) was around 1.5% of all decayed teeth at baseline and 0.5% at follow-up, there is no data about the incidence of such infections between the two time-points and so this study underestimates the burden of inflammation and infection involving the non-vital teeth that was present in the cohort.

The arrest rate achieved by the SDF application in School 1 is consistent with both published reports on caries arrest globally and from within Cambodia [10–14]. Other studies have reported arrest rates of around 50–70% with an annual application of SDF [15]. The children in the present study had an arrest rate of 45.3% at 3-months for the delayed SDF group, and 87.3% for children who had SDF placed at baseline. The findings are consistent with studies examining arrest over time whereby the lesions are more likely to become arrested over time as the lesion becomes more balanced and remineralization is able to occur [12].

Data from this study could be used to estimate clinically relevant differences in outcomes and expected effect sizes which will be important when more robust, step-wedged clinical trials are designed to examine which combinations of therapies should be included in a HPP for addressing the oral health needs of children in Cambodia. The results will be useful for making evidence-based recommendations around which children should receive which interventions and under which circumstances.

It is unlikely that conventional dental care could be funded or sustainably delivered in a publicly-funded program in Cambodia and DTB alone would not be not sufficient to prevent the incidence of pulpally involved teeth. Therefore, the HKC Level 1 package of DTB and SDF might be a realistic first step in moving toward Universal Oral Health Care for primary school-age children in Cambodia. The Level 1 package of care can easily be delivered to all children in a school setting at minimal expense, using both dental and trained non-dental personnel as appropriate. Such an approach would greatly increase access to care, and families would not have to carry the financial and other burdens and barriers associated with accessing conventional dental care.

## Conclusions

This study offers some preliminary evidence that the combination of DTB and SDF can manage most carious lesions in the primary dentition among children in Cambodia, and more importantly help to reduce the incidence of pulpally-involved teeth. Data from this study could be used to estimate clinically relevant differences in outcomes and expected effect sizes which will be important when designing future robust, step-wedged clinical trials.

## Data Availability Statement

The raw data supporting the conclusions of this article will be made available by the authors, without undue reservation.

## Ethics Statement

The studies involving human participants were reviewed and approved by National Ethics Comittee for Health Research, Ministry of Health, Cambodia. Written informed consent to participate in this study was provided by the participants' legal guardian/next of kin.

## Author Contributions

BT drafted the manuscript and performed data analysis. JP, CD, CS, and RT contributed ideas to the manuscript. JP and CD performed editing of the script. CS and RT led delivery of the program in the field. All authors contributed to the article and approved the submitted version.

## Conflict of Interest

The authors declare that the research was conducted in the absence of any commercial or financial relationships that could be construed as a potential conflict of interest.
